# The loss of glycocalyx integrity impairs complement factor H binding and contributes to cyclosporine-induced endothelial cell injury

**DOI:** 10.3389/fmed.2023.891513

**Published:** 2023-02-13

**Authors:** Chia Wei Teoh, Magdalena Riedl Khursigara, Carolina G. Ortiz-Sandoval, Jee Woo Park, Jun Li, Arlette Bohorquez-Hernandez, Valentina Bruno, Emily E. Bowen, Spencer A. Freeman, Lisa A. Robinson, Christoph Licht

**Affiliations:** ^1^Division of Nephrology, The Hospital for Sick Children, Toronto, ON, Canada; ^2^Department of Paediatrics, University of Toronto, Toronto, ON, Canada; ^3^Cell Biology Program, Research Institute, The Hospital for Sick Children, Toronto, ON, Canada; ^4^Institute of Medical Science, University of Toronto, Toronto, ON, Canada; ^5^Division of Paediatric Nephrology, Santobono-Pausilipon Children's Hospital, Naples, Italy; ^6^Bristol Renal, School of Clinical Sciences, University of Bristol, Bristol, United Kingdom; ^7^Department of Biochemistry, University of Toronto, Toronto, ON, Canada; ^8^Department of Laboratory Medicine and Pathobiology, University of Toronto, Toronto, ON, Canada

**Keywords:** thrombotic microangiopathy, calcineurin inhibitors, cyclosporine, endothelium, heparan sulfate, proteoglycans, complement, alternative pathway

## Abstract

**Background:**

Calcineurin inhibitors (CNIs) are associated with nephrotoxicity, endothelial cell dysfunction, and thrombotic microangiopathy (TMA). Evolving evidence suggests an important role for complement dysregulation in the pathogenesis of CNI-induced TMA. However, the exact mechanism(s) of CNI-induced TMA remain(s) unknown.

**Methods:**

Using blood outgrowth endothelial cells (BOECs) from healthy donors, we evaluated the effects of cyclosporine on endothelial cell integrity. Specifically, we determined complement activation (C3c and C9) and regulation (CD46, CD55, CD59, and complement factor H [CFH] deposition) as these occurred on the endothelial cell surface membrane and glycocalyx.

**Results:**

We found that exposing the endothelium to cyclosporine resulted in a dose- and time-dependent enhancement of complement deposition and cytotoxicity. We, therefore, employed flow cytometry, Western blotting/CFH cofactor assays, and immunofluorescence imaging to determine the expression of complement regulators and the functional activity and localization of CFH. Notably, while cyclosporine led to the upregulation of complement regulators CD46, CD55, and CD59 on the endothelial cell surface, it also diminished the endothelial cell glycocalyx through the shedding of heparan sulfate side chains. The weakened endothelial cell glycocalyx resulted in decreased CFH surface binding and surface cofactor activity.

**Conclusion:**

Our findings confirm a role for complement in cyclosporine-induced endothelial injury and suggest that decreased glycocalyx density, induced by cyclosporine, is a mechanism that leads to complement alternative pathway dysregulation *via* decreased CFH surface binding and cofactor activity. This mechanism may apply to other secondary TMAs—in which a role for complement has so far not been recognized—and provide a potential therapeutic target and an important marker for patients on calcineurin inhibitors.

## Introduction

Thrombotic microangiopathies (TMAs) are defined by their common clinical features: microangiopathic hemolytic anemia (MAHA), non-immune thrombocytopenia, and end-organ injury (1–3). TMAs are systemic conditions with the potential for multi-organ involvement, including the kidneys, the brain, the gastrointestinal tract, the respiratory tract, and the skin. Crucial to the development of TMA is injury to the microvascular endothelium. Injuries to the endothelium post its activation lead to excessive platelet and neutrophil recruitment and eventually to thrombus formation, chronic inflammation, and organ failure (1, 4, 5). While complement cascades are critical to mounting appropriate immune responses, the regulation of their products is critical to maintaining host cell integrity, notably for the vascular endothelium. Indeed, the loss of complement regulation favors spontaneous complement activation, resulting in endothelial injury and the formation of (micro-)thrombi (5–7). Complement dysregulation is also increasingly recognized in the pathogenesis of TMAs and is found in patients with various forms of secondary comorbidities (i.e., TMA spectrum) (1, 8–11).

The alternative pathway (AP) of complement is constitutively active (spontaneous tick-over), resulting in a low, but constant, level of circulating C3b in the plasma, which can bind to either host cell or pathogen surfaces. Since C3b is free to coat and disrupt surfaces without distinction, there are regulatory mechanisms that tightly protect host cells from complement-mediated injury, including membrane-associated proteins like membrane cofactor protein (MCP/CD46), decay-accelerating factor (DAF/CD55), and protectin (CD59) as well as the secreted protein complement factor H (CFH), which circulates in human plasma at high (200–300 ug/mL) concentrations (8, 12, 13). The density and localization of these regulatory proteins represent one of the key principles of complement control and are critical to maintaining the integrity of self-surfaces such as the vascular endothelium. Genetic mutations in CD46 or CFH, as well as the expression of anti-CFH autoantibodies, result in excessive complement activation—in particular *via* the alternative pathway—and increase patient susceptibility to develop TMA *via* endothelial injury (14–19). A number of additional mutations in complement (modulator) genes, including C3 itself, complement factor B (CFB), factor I (CFI), and thrombomodulin (THBD/CD141), have also been linked to endothelial cell injury and TMAs. There is, however, variable penetrance described in patient families within a pedigree with complement mutations, implicating a contribution from the environment as being necessary to trigger TMA manifestations in a patient who is genetically susceptible (“multiple-hit” hypothesis) (1, 8, 15). Among the events that precede the onset of TMA, the most relevant are respiratory and gastrointestinal tract infections and pregnancy (16, 20). Secondary TMA can also occur post-transplant when it is associated with antibody-mediated rejection and immunosuppressive medications like calcineurin inhibitors (CNIs) (9, 21–24).

Calcineurin inhibitors (CNIs) such as cyclosporine and tacrolimus are highly effective immunosuppressive agents, which are widely used to prevent allograft rejection in solid organ and hematopoietic stem cell transplantation and to treat autoimmune disorders. Their use is also associated with adverse effects, such as hypertension, nephrotoxicity, vascular injury, and the development of CNI-induced arteriolopathy, which negatively impact patient and allograft survival (25–32). In addition, CNIs are known to trigger post-transplant TMA (28, 29, 31, 33). The possible cause for these adverse effects, in particular TMA, in endothelial injury associated with CNI use, secondary to vasoconstriction-associated ischemia, increased platelet aggregation, and the activation of prothrombotic factors (27).

Evolving evidence suggests an important role for complement dysregulation in the pathogenesis of CNI-induced microvascular endothelial cell injury, which is crucial for the development of TMA (34, 35). Recently, CNI-mediated endothelial injury—in particular in the glomerular capillaries—has been linked to complement activation *in vivo*, and a central role of the complement alternative pathway has been identified (34). The exact mechanism, by which CNI induces complement activation, however, remains poorly understood. Because cyclosporine use is associated with vascular injury, development of TMA, and nephrotoxicity, we examined whether cyclosporine exposure leads to complement-mediated endothelial cell injury and investigated the mechanism by which complement dysregulation is induced in an *in vitro* model utilizing blood outgrowth endothelial cells (BOECs).

## Materials and methods

### Patient samples

BOECs were isolated from the peripheral blood of two healthy adult volunteers. Normal human serum (NHS) was derived from three healthy adult volunteers.

### Blood outgrowth endothelial cells

BOECs were isolated as previously described and cultured in endothelial cell growth medium (cEGM-2: Endothelial Basal Medium 2 [EBM-2] supplemented with growth factors [EGM-2 BulletKit]) (Lonza, Walkersville, MD), 10% fetal bovine serum (FBS) (Sigma-Aldrich, St. Louis, MO), and 1% antibiotic–antimycotic (Gibco, Invitrogen, Life Technologies, Carlsbad, CA) (36, 37). Cells were maintained at 37°C, in an environment with 5% CO_2_. Passages 3–12 were used.

### Cyclosporine treatment and complement fixation on endothelial cells

BOECs grown to confluence were exposed to cyclosporine 10, 20, 50, or 100 μg/ml in media for up to 24 h. Cyclosporine stock solution (Sandimmune IV, Novartis Pharmaceuticals Canada Inc., Dorval, QC, 50 mg/ml) was kindly provided by the SickKids pharmacy. For experiments involving complement fixation on BOECs, cells were sequentially exposed to 50% NHS (1:1 in serum-free media [SFM]) for 30 min. In experiments utilizing an antibody specifically blocking the membrane-anchored complement regulator CD59 (BRIC229, IgG2b, International Blood Group Reference Laboratory, NHS Blood and Transplant, Bristol, UK), cells were incubated for 30 min with the blocking antibody (5 μg/ml) prior to exposure to 50% NHS (36, 38).

### Detection of complement deposition on endothelial cells

C3b and C5b-9 deposition on BOEC surfaces were demonstrated by flow cytometry using a C3c antibody detecting the C3c portion of native C3 and C3b (C3c-FITC conjugated antibody, Abcam, ab4212, 1:50 dilution) and C9 (Complement Technologies Inc, TX, A226, 1:100 dilution). Cells were grown to confluence and exposed to cyclosporine treatment and complement fixation as described. Cells were washed with phosphate-buffered saline (PBS) and incubated with Fixable Viability Dye eFluor780 (eBioscience, San Diego, CA, 1:1,000 dilution reconstituted in PBS) at 4°C for 30 min. For flow cytometry, cells were harvested by scraping and washed with PBS before use (Supplementary material).

### Assessment of Weibel–Palade body mobilization and von Willebrand factor release from endothelial cells

von Willebrand Factor release from BOECs was detected *via* immunofluorescence as described previously (36). BOECs treated with media for 24 h, followed by incubation with anti-CD59 blocking antibody for 30 min and 50% NHS/50% SFM for 30 min, were used as positive control and compared to cells kept in media (negative control). Cells were then washed and fixed with 2% paraformaldehyde and permeabilized with 0.2% Triton in PBS, followed by incubation with rabbit anti-VWF (Dako, Carpinteria, CA, A0082, 1:1,000) and goat anti-VE-cadherin (Santa Cruz Biotechnology, Dallas, TX, sc-6458, 1:250) for 4 h. Alexa Fluor 488- and Alexa Fluor 555-conjugated species-specific secondary antibodies were used at a dilution of 1:1,000. Nuclei of cells were stained with 0.12 μg/ml Hoechst stain (Thermo Fisher Scientific, Waltham, MA) for 5 min.

### Characterization of membrane-anchored complement regulators

To determine the expression level of the membrane-anchored complement regulators MCP/CD46, decay-accelerating factor (DAF/CD55), and CD59 on BOECs, BOEC lysates were utilized for flow cytometry and Western blotting analysis (Supplementary material).

### Detection of CFH binding to endothelial cell surfaces

The binding of CFH to BOEC surfaces was demonstrated by flow cytometry as described previously (39), using purified CFH (CSL Behring, Marburg, Germany) tagged with Alexa Fluor 488 succinimidyl ester (10 μg/mL, Life Technologies) for 1 h at room temperature before being dialyzed overnight in PBS. Cells exposed to 500 mU/mL neuraminidase (MilliporeSigma; N2876) were used as the positive control. Cells were washed two times with PBS and scraped off. Cells were then incubated with Fixable Viability Dye eFluor780 at 4°C for 30 min. They were then washed with PBS and resuspended in 100 μL PBS. Each sample was then incubated with 4 μg of Alexa Fluor 488-tagged CFH for 1 min, after which 500–1,000 μL of Attune focusing fluid (Thermo Fisher Scientific, 4449791) was added and assessed by flow cytometry (Supplementary material).

For immunofluorescence experiments, cells were cultured to a minimum of 80% confluency on collagenized coverslips and exposed to cyclosporine A as described. Cells exposed to 500 mU/mL Neuraminidase for 1 h and 0.5 U/mL Heparinase III (H8891-5UN, Sigma-Aldrich, St. Louis, MO) for 30 min were used as positive controls. Cells were washed and fixed with 4% paraformaldehyde, blocked for 1 h with 3% BSA, followed by incubation with goat anti-Factor H (1:100, Complement Technology Inc., TX; A237) and mouse anti-heparan sulfate (1:50, US Biological Life Sciences, Salem, MA; H1890) overnight at 4°C. Goat Alexa Fluor 488 and Mouse Alexa Fluor 555 secondary antibodies were used, respectively, at a dilution of 1:200 for 1 h at room temperature. The nuclei of the cells were stained with 0.12 μg/ml Hoechst stain (Thermo Fisher Scientific, Waltham, MA) for 5 min. Confocal microscopy was performed as detailed in Supplementary material, and total fluorescence intensity was measured using ImageJ software.

### CFH surface cofactor activity assay

To determine CFH cofactor activity on BOEC surfaces, cells exposed to 500 mU/mL neuraminidase for 1 h (Millipore Sigma; N2876) were used as the positive control. Cofactor activity of surface-bound CFH was detected as previously described (40). Cells were incubated with 10 μg/ml CFH (CSL Behring, Marburg, Germany) at 37°C for 1 h, 10 μg/ml CFI (EMD Millipore Corp., MA, 341280) and with 3.3 μg/ml C3b (EMD Millipore Corp., MA, 204860). The supernatant was collected at baseline and various subsequent time points (up to 180 min), and the samples were transferred to a reduced sample buffer and separated by 10% SDS-PAGE. The appearance of C3b degradation fragments was detected by Western blotting (**Figure 6**). Primary goat anti-C3, 1:1,000 dilution (Complement Technology Inc., TX, A213) with corresponding secondary HRP-conjugated antibody at a dilution of 1:5,000 was used for detection.

### Imaging of glycocalyx

To image the endothelial glycocalyx, an Alexa Fluor 594-conjugated wheat germ agglutinin (WGA, Thermo Fischer Scientific, W11262, dilution 2 μg/ml), anti-heparan sulfate antibody (Abcam, Cambridge, UK, ab23418, 1:100), and peanut agglutinin (PNA, Vector Labs, Ontario, CA, FL-1071-5, 1:200) were used. Cells were cultured to confluence on coverslips and exposed to cyclosporine as described. Cells exposed to 500 mU/mL neuraminidase for 1 h were used as a positive control in WGA and PNA experiments. Cells exposed to 0.5 U/mL Heparinase III (H8891-5UN, Sigma-Aldrich, St. Louis, MO) for 30 min were used as a positive control in heparan sulfate experiments. Cells were incubated with Alexa Fluor 594-conjugated WGA for 5 min on ice and washed two times with ice-cold HBSS, and the coverslips were mounted in a Chamlide magnetic chamber (Life Cell Instrument, Seoul, Korea) and overlaid with media. Confocal microscopy was performed as detailed in Supplementary material, and total fluorescence intensity was measured using ImageJ software. For experiments using anti-heparan sulfate and PNA, cells were washed and fixed with 2% paraformaldehyde, followed by incubation with mouse anti-heparan sulfate (1:100) and anti-PNA (1:100) for 1 h. Alexa Fluor 488-conjugated species-specific secondary antibodies were used at a dilution of 1:1,000. Nuclei of cells were stained with 0.12 μg/ml Hoechst stain (Thermo Fisher Scientific, Waltham, MA) for 5 min.

### Statistics

Figures were generated with GraphPad Prism (Version 6.0c; GraphPad Software, La Jolla, CA) and displayed as the mean and standard deviation. Statistical analysis was performed *via* paired *t*-test or two-way ANOVA with *post-hoc* analysis. A *p* < 0.05 was considered statistically significant. In the figure legends, *p*-values are presented as follows: ^*^p < 0.05, ^**^p < 0.01, ^***^p < 0.001, and ^****^p < 0.0001.

## Results

### Cyclosporine causes endothelial cell injury and complement deposition

The use of cyclosporine is associated with a vascular injury in pathophysiological situations. We, therefore, tested whether cyclosporine treatment of cultured BOECs caused endothelial cell toxicity using an established lactate dehydrogenase (LDH) assay for lytic cell death. We found that cyclosporine caused cytotoxicity of BOEC cultures in a dose- and time-dependent fashion (Supplementary Figure S1). Specifically, the acute (1 h) treatment of BOECs with low concentrations (<50 μg/mL) of cyclosporine did not cause cell lysis, while a 24-h treatment of the cells with cyclosporine used above 250 μg/mL led to lysis of nearly the entire culture. Intermediate concentrations of cyclosporine (50 μg/mL) caused ~60% of the cells to rupture, and lower concentrations of 10 μg/ml did not lead to any detectable LDH release (Supplementary Figure S1). We, therefore, chose to treat BOECs within the range of 10 μg/mL (non-lethal) and 50 μg/mL (~half-maximal lysis) concentrations of cyclosporine in subsequent experiments.

**Figure 1:**
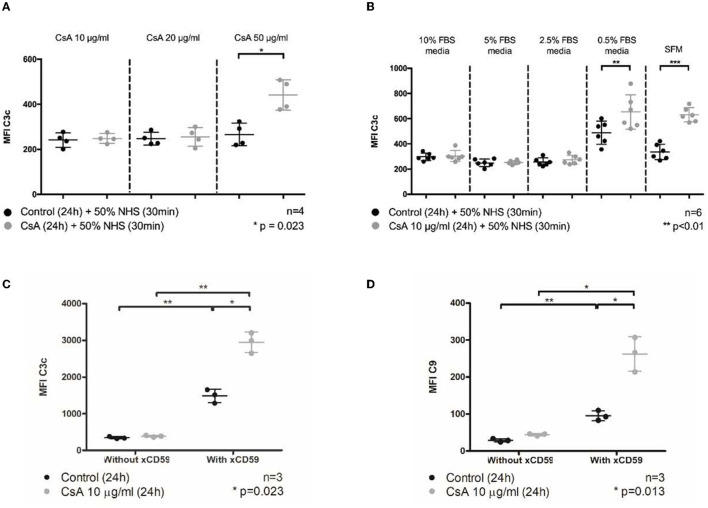
Cyclosporine causes complement deposition on endothelial cell surfaces, enhanced by serum starvation and anti-CD59 sensitization. Blood outgrowth endothelial cells (BOECs) were incubated in cyclosporine (CsA) for 24 h, followed by 50% NHS for 30 min. Unless specified, cyclosporine was reconstituted with media/10% FBS. C3c and C9 deposition on BOEC surfaces was detected by flow cytometry. Non-viable cells were excluded from analysis with Fixable Viability Dye eFluor 780. **(A)** Incubating BOECs with cyclosporine 50 μg/ml resulted in significantly higher C3c deposition (*n* = 4, *p* = 0.023, paired, two-tailed *t*-test). **(B)** Incubating BOECs with cyclosporine 10 μg/ml reconstituted in media supplemented with decreasing amounts of FBS resulted in significantly higher C3c deposition (*n* = 6, *p* < 0.01, paired, two-tailed *t*-test). **(C)** Addition of anti-CD59 antibody enhanced cyclosporine-induced C3c deposition. Incubation of BOECs with cyclosporine 10 μg/ml for 24 h, followed by anti-CD59 antibody incubation for 30 min, prior to 50% NHS for 30 min caused a significantly increased C3c deposition (*n* = 3, *p* = 0.023, two-way ANOVA, Sidak's multiple comparison test). **(D)** Addition of anti-CD59 antibody also enhanced cyclosporine-induced C9 deposition (*n* = 3, *p* = 0.013, two-way ANOVA, Sidak's multiple comparison test). In keeping with previous data, no increase in C9 deposition was detected when BOECs were incubated with media or cyclosporine 10 μg/ml alone. This *** signifies the degree of statistical significance as denoted by the *p* value in the figure and in the “Statistics” section in Materials & Methods.

To that end, confluent monolayers of BOECs were treated with these concentrations of cyclosporine in medium containing 10% fetal bovine serum (FBS) for 24 h and subsequently exposed to 50% NHS in serum-free medium (SFM) for 30 min as established in Supplementary Figure S1. Under these conditions, we found that the treatment of BOECs with 50 μg/ml of cyclosporine caused a significant increase in complement C3 deposition (Figure 1A, MFI: cyclosporine 50 μg/mL 441.1 ± 67.1 vs. control 265.8 ± 50.1, *n* = 4, *p* = 0.023). Using lower doses of cyclosporine (10 μg/ml), we determined that the increased deposition of C3c was enhanced in the absence of serum. Factors in the serum prevented C3c deposition on the cyclosporine-treated BOEC cultures: >2.5% FBS prevented C3c deposition, while at <0.5% FBS, significantly increased C3c was detected on the surface of cyclosporine-treated cells (Figure 1B, MFI: cyclosporine 10 μg/mL in serum-free media 622.5 ± 32.72 vs. control 343.1 ± 65.84, *n* = 6, *p* < 0.01).

Inhibiting the function of CD59, a membrane-anchored complement regulator, is an established means of sensitizing complement fixation on endothelial cells. Blocking CD59 with antibodies has the dual effect of complement induction mainly *via* sensitization (classical pathway) but also through complement amplification (alternative pathway) (36, 38, 41–43). We were interested to examine whether cyclosporine had general effects on the membrane topology that impact C3c deposition or if its effect was *via* CD59. Using the same flow cytometry approach used in Figure 1B, we found that blocking CD59 indeed led to a large increase in C3c associated with the endothelial cells (Figure 1C). However, cyclosporine treatment further increased C3 deposition ~2-fold beyond the level achieved by blocking CD59 alone. This effect was also observed for C5b-9 to an even greater extend fold increase (Figure 1D). Thus, BOECs treated with cyclosporine had a dose-dependent injury concomitant with increased complement deposition that could be enhanced by the removal of serum or complement regulators.

### Cyclosporine induces von Willebrand factor release from endothelial cells

Weibel–Palade bodies **(**WPBs) are endothelial storage granules containing pro-hemostatic and pro-inflammatory molecules, including VWF, P-selectin, interleukin-8, endothelin-1, and angiopoietin-2 (44–46). As previously demonstrated by us and others, WPBs are exocytosed upon endothelial cell injury and activation to release their contents, which potentiates inflammatory responses, vascular leakage, and leukocyte adhesion (36, 45, 47). Given that cyclosporine resulted in endothelial cell injury and complement deposition, we hypothesized that cyclosporine treatment may also lead to the endothelial release of VWF.

**Figure 2:**
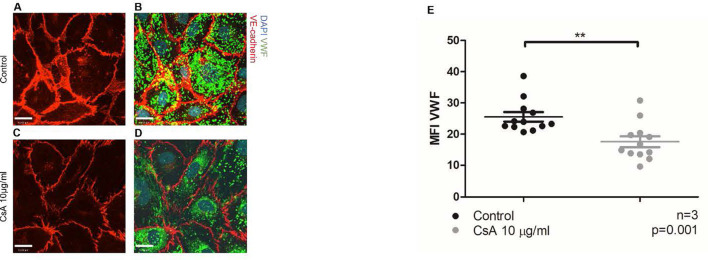
Cyclosporine causes endothelial cell release of Weibel–Palade bodies and its contents. **(A–E)** Von Willebrand factor (VWF) release from blood outgrowth endothelial cells (BOECs) was detected *via* immunofluorescence. Control BOECs were treated with media for 24 h. The experimental condition was with BOECs treated with cyclosporine (CsA) 10 μg/ml for 24 h. BOECs were fixed with 2% paraformaldehyde and permeabilized with 0.2% Triton in phosphate-buffered saline, followed by incubation with rabbit anti-VWF (1:1,000) and goat anti-VE-cadherin (1:250) for 4 h. Alexa Fluor 488 (green)- and 555 (red)-conjugated species-specific secondary antibodies were used at dilution of 1:1,000. Nuclei were stained with 0.12 μg/ml Hoechst stain for 5 min. Images were taken using an IX81 inverted microscope (Olympus Corp., Tokyo, Japan) with a 60/1.35 oil immersion objective and a C9100-13 back-thinned EM-CCD camera (Hamamatsu Photonics, Hamamatsu City, Shizuoka Pref., Japan) with a CSU X1 spinning disk confocal scan head (Yokogawa, Yokogawa Canada Inc., AB). Bar = 22 μm. Treatment with cyclosporine 10 μg/ml for 24 h led to less intracellular VWF and less intense staining of VE-cadherin **(A–E)** (*n* = 3, **p = 0.001, two-tailed *t*-test).

Using the previously established protocol, we first showed that complement activation indeed caused the release of intracellular VWF (Supplementary Figure S2—positive control using anti-CD59 sensitization) (36). We then found that BOECs treated with cyclosporine 10 μg/mL had less intense staining of intracellular VWF (Figure 2). Taken together, our results showed that cyclosporine induces VWF release from BOECs.

### Cyclosporine treatment leads to the increased expression of membrane-associated complement regulators

**Figure 3:**
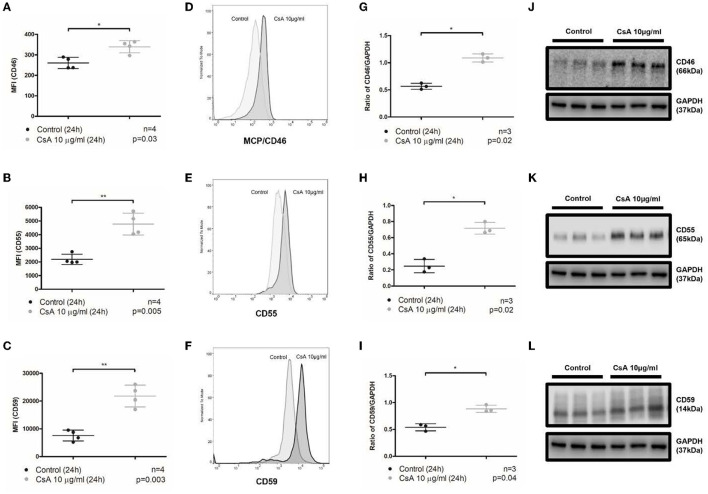
Cyclosporine causes increased expression of membrane-bound complement regulators MCP/CD46, DAF/CD55, and CD59 on endothelial cell surfaces. **(A–F)** Blood outgrowth endothelial cell (BOEC) surface membrane-bound complement regulators detected by flow cytometry. Non-viable cells were excluded from analysis with Fixable Viability Dye eFluor 780. Graphical summary of median fluorescence intensity (MFI) from separate experiments **(A–C)** with representative histograms **(D–F)**. Compared to control, incubating BOECs in cyclosporine (CsA) 10 μg/ml for 24 h resulted in increased expression of membrane-bound complement regulators on BOEC surfaces: **(A, D)** MCP/CD46 (*n* = 4, *p* = 0.03, paired, two-tailed *t*-test), **(B, E)** DAF/CD55 (*n* = 4, *p* = 0.005, paired, two-tailed *t*-test), and **(C, F)** CD59 (*n* = 4, *p* = 0.003, paired, two-tailed *t*-test). **(G–L)** Membrane-bound complement regulators MCP/CD46, DAF/CD55, and CD59 in cell lysates by Western blot. Graphical summary of mean gray area of specified protein band normalized to loading control from separate experiments **(G–I)** and Western blots **(J–L)**. Compared to media, incubating BOECs in cyclosporine 10 μg/ml for 24 h resulted in increased BOEC protein content of membrane-bound complement regulators: **(G, J)** MCP/CD46 (*n* = 3, *p* = 0.02, paired, two-tailed *t*-test), **(H, K)** DAF/CD55 (*n* = 3, *p* = 0.02, paired, two-tailed *t*-test), and **(I, L)** CD59 (*n* = 3, *p* = 0.04, paired, two-tailed *t*-test). This * and ** signifies the degree of statistical significance as denoted by the p value in the figure and in the “Statistics” section in Materials & Methods.

The regulation of the alternative pathway of complement activation is executed by a combination of fluid-phase (CFH and CFI) and membrane-bound regulators (mainly MCP/CD46, DAF/CD55, and CD59) that maintain the balance between complement activation and inhibition (8, 13). Given that cyclosporine caused an increase in complement activation on the surface of BOECs, it was conceivable that cyclosporine decreased the expression of membrane-bound complement regulators. We, therefore, assessed the expression of MCP/CD46, DAF/CD55, and CD59 on the surface of BOECs after their treatment with cyclosporine using flow cytometry, and the total cell expression of these regulators by probing cell lysates with Western blotting. We found that treatment of the BOECs with low concentrations (10 μg/mL) of cyclosporine resulted in the increased surface and total cell expression of MCP/CD46, DAF/CD55, and CD59 (Figure 3). Incubation with cyclosporine at higher concentrations of cyclosporine (50 μg/mL) resulted in a similar effect (data not shown). Thus, the increased complement deposition on the surface of cyclosporine-treated cells was not the result of the lost expression of membrane-bound complement regulators.

### Cyclosporine treatment leads to impaired CFH binding and regulation on endothelial cells

Since enhanced complement deposition induced by cyclosporine occurred in the context of *increased* expression of membrane-bound complement regulators, we hypothesized that cyclosporine may instead impair CFH-mediated complement regulation. CFH is the central circulating alternative pathway inhibitor, which competitively prevents C3b deposition on cell surfaces, acts as a cofactor to CFI to cleave surface-bound C3b, and accelerates the decay of the C3bBb complex (48–50). To exert these functions, CFH is known to be closely associated with endothelial surfaces *via* its multiple glycosaminoglycan/sialic acid-binding domains (51–55).

**Figure 4:**
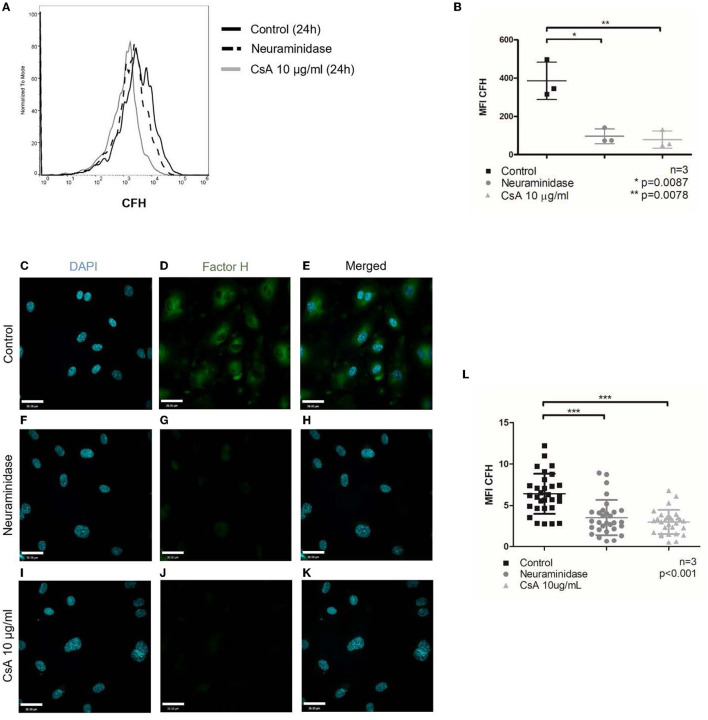
Cyclosporine causes reduced complement factor H binding on surfaces of endothelial cells. Alexa Fluor 488-conjugated complement factor H (CFH) binding on blood outgrowth endothelial cell (BOEC) surface was assessed by flow cytometry **(A, B)**. Non-viable cells were excluded from analysis with Fixable Viability Dye eFluor 780. Representative histogram **(A)** and graphical summary of mean fluorescence intensity from separate experiments **(B)**. Compared to control (no cyclosporine), incubating BOECs in cyclosporine (CsA) 10 μg/ml for 24 h resulted in reduced CFH binding on BOEC surface (*n* = 3, **p = 0.0078, paired, two-tailed *t*-test). Treatment with neuraminidase 500 mU/ml, which cleaves sialic acid groups from glycoproteins, also resulted in reduced CFH binding on BOEC surface (*n* = 3, *p = 0.0087, paired, two-tailed *t*-test). CFH was also assessed by immunofluorescence **(C–L)**. Representative images **(C–K)** and mean fluorescence intensity from three sets of experiments with 10 representative images taken per condition (each dot represents 1 image) were measured with ImageJ and summarized **(L)**. Compared to control, incubating BOECs in CsA 10 μg/ml for 24 h resulted in reduced CFH binding on BOEC surface (*n* = 3, ***p < 0.001, paired, two-tailed *t*-test)

To test whether cyclosporine impaired CFH binding, we pre-treated BOECs with cyclosporine and then assessed the ability of the cells to secure Alexa Fluor 488-conjugated CFH from the culture medium. The Alexa Fluor 488-labeled CFH was added for 1 min to live cells before their analysis by flow cytometry. We found that incubation of BOECs with cyclosporine at 10 μg/mL for 24 h caused a significant reduction in CFH binding (Figures 4A, B, MFI control 386.3 ± 97.8 vs. cyclosporine 10 μg/mL 78.3 ± 45.8, *n* = 3, *p* = 0.0078). A brief (1 h) treatment of the cells with neuraminidase used at 500 mU/mL, an enzyme that cleaves terminal sialic acid groups from glycoproteins, was used as a positive control. The functionality of neuraminidase in cleaving sialic acid was confirmed by live imaging with wheat germ agglutinin (WGA; see the section below) and by CFH binding. Removal of sialic acids inhibited CFH binding to the endothelium to nearly the same extent as cyclosporine treatment (Figure 4B). This reduction in CFH binding on cells treated with cyclosporine 10 μg/mL for 24 h was also confirmed by immunofluorescence (Figures 4C–L, MFI control 6.43 ± 0.44 vs. cyclosporine 10 μg/mL 3.03 ± 0.26, *n* = 3, *p* < 0.001).

**Figure 5:**
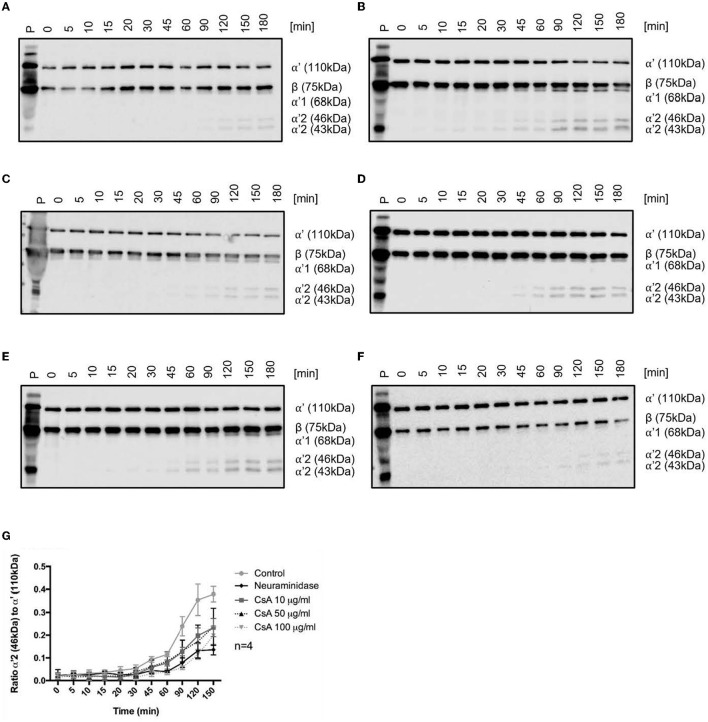
Cyclosporine causes impaired complement factor H regulation on surfaces of endothelial cells. **(A–F)** Cyclosporine (CsA) leads to impaired complement factor H (CFH) surface cofactor activity detected by a CFH surface cofactor activity assay. Blood outgrowth endothelial cells (BOECs) were incubated with C3b 3.3 μg/ml and complement factor I (CFI) 10 μg/ml at 37 degrees Celsius, with or without pre-incubation with CFH 10 μg/ml at 37 degrees Celsius. The appearance of C3b degradation fragments was analyzed by Western blotting (representative Western blots are shown in **(A–F)**. **(A)** Endogenous cofactor activity on BOEC without CFH. BOECs were incubated with C3b and CFI at 37 degrees Celsius. Degradation products (α'68, α'46, and α'43 kDa fragments of the C3b α' chain) were detectable after 90 min and increased with time. **(B)** Cofactor activity of CFH on the surface of BOEC. BOECs were pre-incubated with CFH for 1 h at 37 degrees Celsius and thoroughly washed, prior to incubation with C3b and CFI at 37 degrees Celsius. Degradation products were detectable after 15 min. **(C)** Cofactor activity of CFH on the surface of neuraminidase-treated BOEC. Neuraminidase cleaves sialic acid groups from cell surfaces. BOECs were pre-incubated with neuraminidase 500 mU/ml for 1 h followed by CFH for 1 h at 37 degrees Celsius, prior to being thoroughly washed and incubated with C3b and CFI at 37 degrees Celsius. Degradation products were detectable after 60 min. **(D–F)** Cofactor activity of CFH on the surface of cyclosporine-treated BOEC. BOECs were pre-incubated with **(D)** cyclosporine 10 μg/ml, **(E)** cyclosporine 50 μg/ml, and **(F)** cyclosporine 100 μg/ml for 24 h. They were then incubated with CFH for 1 h at 37 degrees Celsius, and C3b degradation products were detectable: **(D)** cyclosporine 10 μg/ml after 45 min, **(E)** cyclosporine 50 μg/ml after 45 min, and **(F)** cyclosporine 100 μg/ml after 90 min. These results suggest that cyclosporine causes impaired CFH binding and regulation on surfaces of BOECs. **(G)** Graphical presentation of CFH surface cofactor activity assay experiments. For statistical analysis, we formulated a ratio of the mean gray value of the α'2 46 kDa band with the mean gray value of the α'110 kDa band. An increased ratio indicates that the α' chain was cleaved into its split products, indicative of C3b inactivation. There was a significant reduction in CFH cofactor activity on the surfaces of BOECs treated with cyclosporine when compared with control (*n* = 4, *p* < 0.005 for control vs. cyclosporine 10 μg/ml from 90 min onwards; *p* < 0.04 for control vs. cyclosporine 50 μg/ml from 90 min onwards; *p* < 0.03 for control vs. cyclosporine 100 μg/ml from 45 min onwards; *p* < 0.001 for control vs. neuraminidase 500 mU/ml from 60 min onwards paired, two-tailed *t*-test).

Locally concentrating CFH to the membrane of the vascular endothelium is critical for the protection of the membrane from complement deposition. The activity of the CFH, once docked to the endothelial surface, can subsequently be measured by assays that determined the degradation of complement. We assessed the functional consequences of the cyclosporine-induced reduction in CFH binding to BOECs by employing a previously established CFH surface cofactor activity assay (40). In this assay, endothelial cell-bound CFH was used as the sole source of CFH. The incubation of C3b with CFI and CFH results in C3b degradation with the appearance of C3b fragments with molecular weights of 68 kDa (C3b α'1), 43/46 kDa (C3b α'2), all of which can be detected *via* the same Western blotting approach. We first assessed the endogenous cofactor activity of the membrane-bound complement regulator MCP/CD46 in the absence of CFH when exposed to media (control) and various concentrations of cyclosporine (10, 50, and 100 μg/mL). Degradation products were detectable after 90 min, with no detectable significant differences between cyclosporine concentrations (Figure 5A, Supplementary Figures 3A, C, E, G, I). Pre-incubation of BOECs with CFH resulted in the appearance of C3b degradation products after 15 min (Figure 5B), demonstrating the expected significantly higher cofactor activity of CFH on endothelial surfaces. However, when BOECs were pre-incubated with neuraminidase followed by incubation with CFH and subsequently with C3b and CFI in the absence of additional CFH, degradation products were detectable only after 60 min. This result was in keeping with a lack of surface CFH in cells devoid of sialic acids (Figure 5C).

We then assessed the effect of cyclosporine exposure on the cofactor activity of surface-bound CFH. BOECs exposed to increasing doses of cyclosporine (10, 50, and 100 μg/mL for 24 h) demonstrated a dose-dependent decrease in CFH cofactor activity as evidenced by the later appearances of C3b degradation products: cyclosporine 10 μg/mL after 45 min, cyclosporine 50 μg/mL after 45 min, and cyclosporine 100 μg/mL after 90 min (Figures 5D–F). Taken together, we found decreased cofactor activity of CFH on BOECs pre-treated with cyclosporine (Figure 5G).

### Cyclosporine treatment weakens the endothelium glycocalyx with reduced CFH surface binding

**Figure 6:**
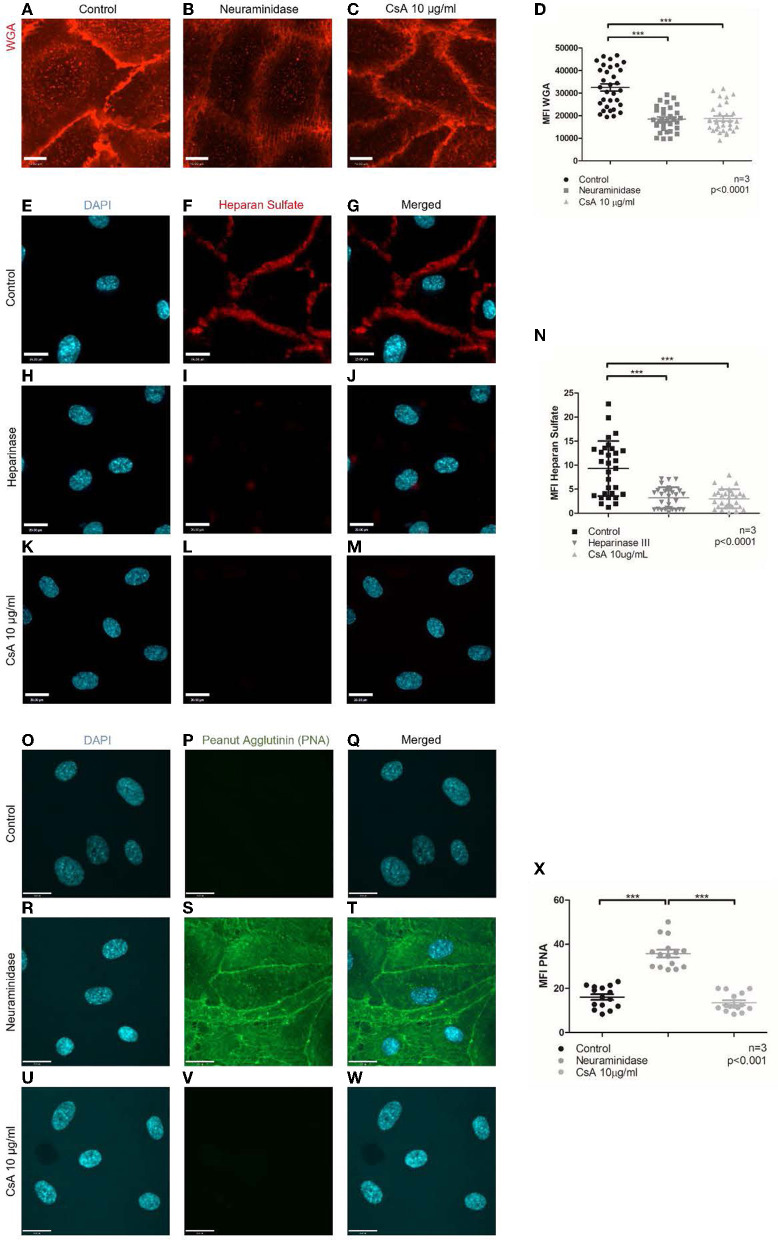
Cyclosporine causes breakdown of endothelial cell glycocalyx. **(A–D)** Live cell imaging of blood outgrowth endothelial cell (BOEC) glycocalyx with Alexa Fluor 594-conjugated wheat germ agglutinin (WGA). When compared to BOECs treated with **(A)** media for 24 h (control), BOECs treated with **(B)** neuraminidase 500 mU/ml for 1 h and **(C)** cyclosporine (CsA) 10 μg/ml for 24 h were less intensely stained with WGA. Mean fluorescence intensity from three sets of experiments with 10 representative images taken per condition (each dot represents 1 image) was measured with ImageJ and summarized in **(D)**. There was a significant reduction in mean fluorescence intensity of WGA staining on BOECs treated with cyclosporine (*n* = 3, ***p < 0.0001, two-tailed *t*-test). Bar = 22 μm. **(E–X)** Heparan sulfate and PNA were detected *via* immunofluorescence. Control BOECs were treated with media for 24 h, BOECs treated with neuraminidase 500 mU/ml for 1 h were used as positive control for PNA experiments, and BOECs treated with heparinase III 0.5 U/ml for 30 min were used as positive control for heparan sulfate experiments. BOECs were fixed with 2% paraformaldehyde, followed by incubation with mouse anti-heparan sulfate (green) and anti-PNA (green). Images were taken using an IX81 inverted microscope (Olympus Corp., Tokyo, Japan) with a 60/1.35 oil immersion objective and a C9100-13 back-thinned EM-CCD camera (Hamamatsu Photonics, Hamamatsu City, Shizuoka Pref., Japan) with a CSU X1 spinning disk confocal scan head (Yokogawa, Yokogawa Canada Inc., AB). Bar = 22 μm. When compared to control, treatment with cyclosporine 10 μg/ml for 24 h led to reduction in heparan sulfate **(E–N)** (*n* = 3, 10 representative images taken per condition, ***p < 0.0001, two-tailed *t*-test) but similar staining with PNA **(O–X)** (*n* = 3, 5 representative images taken per condition, ***p < 0.001, two-tailed *t*-test). Cells treated with neuraminidase 500 mU/ml (positive control) confirmed increased PNA staining **(R–T)**.

CFH has been reported to bind to endothelial surfaces *via* its glycosaminoglycan/sialic acid-binding domains (51–55). Since removing sialic acids with neuraminidase ablated CFH binding to the same extent as cyclosporine treatment, we assessed whether cyclosporine exerted its inhibitory effects on CFH binding *via* remodeling of the glycocalyx. We first stained glycans/polysaccharides containing sialic acid and N-acetyl-D-glucosamine using the lectin wheat germ agglutinin (WGA) conjugated to Alexa Fluor 594. Of note, we imaged the cells live as fixation resulted in a dramatic decrease in overall fluorescence. To prevent endocytosis of the lectin, incubation with Alexa Fluor 594-WGA was performed in the cold (4°C). We determined a decrease in Alexa Fluor 594-WGA staining in BOECs treated with neuraminidase used at 500 mU/mL for 1 h, with conditions identical to those that inhibited CFH binding (Figures 5C, 6A, B: MFI neuraminidase 18,459 ± 6,154 vs. control 32,525 ± 8,990, *p* < 0.0001). Treatment with cyclosporine at 10 μg/mL also resulted in less intense staining with Alexa Fluor 594-WGA when compared to control (Figures 6C, D: MFI cyclosporine 10 μg/mL 18,752 ± 6,154 vs. control 32,525 ± 8,990, *p* < 0.0001). The decrease in the WGA signal in cyclosporine was more apparent in the clusters on the apical surface of the endothelial cells and less visible at cell–cell junctions (Figures 6A, C).

We further assessed whether cyclosporine had additional effects on the endothelial glycocalyx, specifically on the surface density of heparan sulfates. Heparan sulfates are covalently attached to the proteoglycans process in the Golgi apparatus (e.g., syndecans and glypicans). These side chains can be detected by immunostaining: While the polysaccharides may not be immunogenic on their own, in the context of proteoglycans, good antibodies have been generated and made commercially available. We, therefore, immunostained non-permeabilized control or cyclosporine-treated endothelial cells with anti-heparan sulfate antibodies. When compared to control, treatment with cyclosporine used at 10 μg/mL resulted in an ~60% decrease in the intensity of heparan sulfate per cell (Figures 6E–N: MFI cyclosporine 10 μg/mL 3.01 ± 0.36 vs. control 9.30 ± 1.04, *p* < 0.0001). Heparinase III, a polysaccharide lyase that degrades heparan sulfate, was used as the positive control, which led to a similar decrease in the intensity of heparan sulfate (Figures 6H–J, N: MFI heparinase III 3.19 ± 0.41 vs. control 9.30 ± 1.04, *p* < 0.0001). Treatment with cyclosporine 10 μg/mL and heparinase III led to a similar decrease in CFH (Supplementary Figure S4: MFI cyclosporine 10 μg/mL 3.03 ± 0.26 vs. heparinase III 4.11 ± 0.20 vs. control 6.43 ± 0.44, *p* < 0.0001).

Finally, the modifications to the glycocalyx upon cyclosporine treatment could be the result of overactive hydrolases (i.e., glycosidases or proteases) or the result of mistrafficking and expression of proteoglycans and glycoproteins. To determine whether surface glycoproteins in cyclosporine-treated cells were devoid of sialic acids, we used a lectin, peanut agglutinin (PNA), that recognizes exposed, terminal galactose sugars. We found that cyclosporine-treated cells did not have cleaved sialic acids from surface glycoproteins as evidenced by no PNA signal observed on the surface of the cells (Figures 6 O–X: MFI cyclosporine 10 μg/mL 13.6 ± 4.1 vs. control 16.1 ± 4.9, *p* = ns). As a positive control, we showed increased PNA staining in BOECs treated with neuraminidase (Figures 6R–T, X: MFI neuraminidase 35.8 ± 6.7 vs. control 16.1 ± 4.9, *p* < 0.001). The findings with PNA were also confirmed on flow cytometry (Supplementary Figure S5).

Taken together, our findings suggest that cyclosporine treatment results in endothelial glycocalyx breakdown *via* the loss of surface glycoproteins and heparan sulfates, which leads to impaired CFH surface binding.

## Discussion

Calcineurin inhibitor use is associated with acute and chronic tubulo-interstitial, arteriolar, and glomerular injury (27, 32). While possible mechanisms of injury relate to vasoconstriction-associated ischemia, increased platelet aggregation, activation of prothrombotic factors, and disruption of vascular endothelial growth factor (VEGF) regulation of angiogenesis (56), evolving evidence also suggests the involvement of the complement system (34). The association between CNI use and the development of TMA in patients (28, 30, 31) and the observation of complement deposition in areas of endothelial injury in kidney biopsy specimens affected by CNI toxicity hint the involvement of complement (57). Animal models of CNI toxicity implicate the complement system and offer explanations of how further complement-mediated injury can be propagated (34, 35). However, the exact mechanism by which CNIs induce complement activation is still unknown.

Our findings shed light on the pathogenesis of CNI toxicity and specifically identify complement activation on the vascular endothelium as a mechanism. To our knowledge, we are the first to establish an *in vitro* model utilizing BOECs to study the effect of cyclosporine and complement activation on endothelial cells. We found that cyclosporine treatment causes complement deposition and endothelial cell injury, which results in VWF release from Weibel–Palade bodies.

Our findings suggest a role for complement-mediated endothelial cell injury induced by cyclosporine and, for the first time, implicate CFH surface dysregulation in cyclosporine-induced complement activation on endothelial cells. CFH, a plasma protein acting as a cofactor to CFI-mediated cleavage of C3b, must recognize and bind to endothelial cell glycocalyx glycosaminoglycans and terminal sialic residues *via* short consensus repeats (SCRs) 6–8 and 19–20 (48, 51–54). Adapting a previously described flow cytometry protocol of quantifying the binding of CFH and a previously established method of assessing the surface cofactor activity of CFH (39, 40), we found that cyclosporine treatment led to decreased CFH binding to endothelial cell surfaces and impaired CFH surface cofactor activity. In these assays, we also treated BOECs with neuraminidase to test whether the absence of sialic acid on the glycocalyx of endothelial cells affected the binding and surface cofactor activity of CFH. The neuraminidase used (derived from Clostridium perfringens) primarily targets sialic acids in α2,3 (to a lesser extent α2,6 and α2,8) configuration and can cleave terminal sialic acid from O-linked glycans, N-linked glycans, and glycolipids. Of particular interest, we found that neuraminidase treatment led to a similar impairment of CFH surface binding and cofactor activity, suggesting the possibility that cyclosporine affects CFH binding to endothelial cell surfaces by reduction of the glycocalyx.

Utilizing live cell imaging of endothelial cells stained with wheat germ agglutinin (WGA) that binds to sialic acid and N-acetylglucosaminyl residues within the endothelial cell glycocalyx, we found that cyclosporine and neuraminidase treatment significantly diminished the endothelial cell glycocalyx. Furthermore, we found that cyclosporine-induced endothelial cell glycocalyx breakdown occurred mainly through the loss of heparan sulfate. Taken together, these findings suggest that cyclosporine treatment leads to the shedding of heparan sulfate in the endothelial cell glycocalyx, leading to impaired CFH recognition of and binding to host endothelial cell surfaces, which impairs its surface regulation of the alternative pathway. The inability of CFH to inactivate C3b covalently bound to endothelial cell surfaces results in an uninhibited amplification loop that allows for the full activation of the complement cascade. This mechanism leading to alternative pathway dysregulation by CFH could potentially be generalized to other forms of TMA where endothelial cell glycocalyx injury is involved.

Contrary to our initial hypothesis, we found that cyclosporine treatment caused increased expression of the surface membrane-bound complement regulators MCP/CD46, DAF/CD55, and CD59, a possible compensatory cellular response to cyclosporine treatment and the resultant impaired CFH regulation of the alternative pathway. MCP/CD46 aids in the inactivation of C3b as a cofactor in the CFI-catalyzed cleavage of C3b, DAF/CD55 accelerates the disintegration of the C3 and C5 convertases, and CD59 prevents the formation of the membrane attack complex (C5b-9) by binding to C8. The failure of CFH to bind to endothelial cell surfaces and exert its function that is induced in our model by cyclosporine leads to an increased C3b load, which, when not tightly regulated, will be amplified with the formation of the C3 convertases and even more C3b, eventually leading to the activation of the terminal pathway. In this context, we speculate that increasing the expression of the other complement regulatory armamentarium would be in the host endothelial cells' best survival interest.

When cyclosporine was reconstituted in standard endothelial growth medium, there was increased complement deposition (C3 and C9) with cyclosporine 50 μg/ml or higher. When reconstituted in serum-free media, increased complement deposition occurred with cyclosporine 10 μg/ml, suggesting that serum-starved BOECs were more susceptible to cyclosporine-induced complement deposition. Incubating cells with an anti-CD59 blocking antibody, an established model to induce complement deposition on endothelial cells (36, 41–43), led to further enhancement of cyclosporine-induced complement deposition on endothelial cells. Given the ~2-fold increase in surface expression of CD59 after exposure to cyclosporine, the fact that the anti-CD59 is a monoclonal IgG2b antibody—an isotype that activates complement *via* the classical pathway—and the fact that anti-CD59 inhibits the action of the surface-bound complement regulator CD59, the increased complement deposition on endothelial cells induced by cyclosporine is likely due to anti-CD59 antibody-initiated activation of the classical pathway, exacerbated by a reduced capacity to regulate the amplification propagated *via* the alternative pathway (36, 58).

Within our model, we found an optimal balance of endothelial cell survival and CNI effect with cyclosporine doses between 10 and 100 μg/ml for up to 24 h. In the clinical setting, the therapeutic target trough range for cyclosporine is maintained between 100 and 400 ng/ml but varies depending on the indication of its use, the type of transplant, the use of concomitant immunosuppression, and time post-transplant. Suggested target 2-h post-dose levels could be as high as 2 μg/ml (59). *In vitro* experimental studies of cyclosporine effect on various endothelial cell lines used a wide range of drug concentrations ranging from 0.1 μg/ml to 4000 μg/ml over varying exposure durations (up to 72 h) (25, 34, 60–64). Although the levels of cyclosporine maintained clinically are lower than those used in experimental *in vitro* studies, they are not directly comparable. It is a limitation of *in vitro* models of disease, and the differences reflect different susceptibility of various endothelial cell lines and inter-species differences. The duration of exposure used in *in vitro* models is also limited to 24–72 h, whereas many patients are on life-long immunosuppression. To our knowledge, we are the first to study the effect of cyclosporine utilizing BOECs.

**Figure 7:**
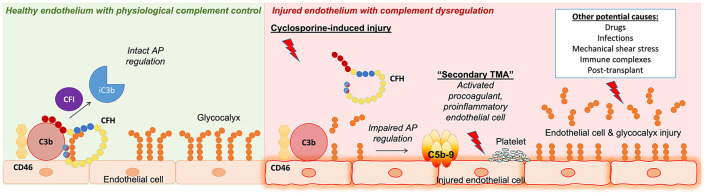
Summary of key findings and concepts presented in our work. **(Left)** Healthy endothelium with physiological complement control where the regulation of the complement alternative pathway (AP) is intact. Complement factor H (CFH) acts as a cofactor with complement factor I (CFI) and membrane cofactor protein (CD46) to inactivate C3b on endothelial cell surfaces. **(Right)** Injured endothelium with complement dysfunction. Our work presented in this study identified a role for complement in cyclosporine-induced endothelial cell injury. We showed that endothelial cells exposed to cyclosporine had decreased glycocalyx density, leading to complement AP dysregulation *via* decreased CFH surface binding and cofactor activity. This mechanism of endothelial cell and glycocalyx injury leading to complement AP dysfunction could potentially be applicable to other forms of secondary thrombotic microangiopathy (TMA).

In conclusion, we found that cyclosporine leads to injury of the endothelial cell glycocalyx and breakdown of heparan sulfate that negatively impacts CFH regulation of the alternative pathway of complement *via* decreased CFH binding to the endothelial cell surface (Figure 7). Enhanced susceptibility to complement-mediated injury secondary to impaired regulation of the alternative pathway might represent a shared mechanism of endothelial injury applicable to various forms of (secondary) TMA, including those caused by toxic agents, mechanical stress, and autoantibodies, which warrants further elucidation.

## Data availability statement

The original contributions presented in the study are included in the article/Supplementary material. Further inquiries can be directed to the corresponding author.

## Ethics statement

The study was approved by the Research Ethics Board of the Hospital for Sick Children (SickKids), Toronto, ON. Signed written informed consent was obtained from all volunteers whose samples were used in the study. The study was performed in keeping with the Declaration of Helsinki.

## Author contributions

CWT designed and coordinated the project, performed experiments, interpreted the results, and wrote the initial and subsequent revised versions of the manuscript. MR designed the project, performed experiments, interpreted the results, and reviewed the manuscript. CO-S performed experiments, interpreted the results, and reviewed the manuscript. SF designed experiments, interpreted the results, and reviewed the manuscript. JP, JL, AB-H, VB, and EB performed experiments and reviewed the manuscript. LR interpreted the results and reviewed the manuscript. CL designed and coordinated the project, interpreted the results, and reviewed the manuscript. All authors contributed to the article and approved the submitted version.

## References

[1] Noone DG Riedl M Pluthero FG Kahr WHA Bowman ML James P. The association of von Willebrand factor and the alternative complement pathway in atypical hemolytic uremic syndrome - complement amplifying or protective? J Am Soc Nephrol. (2014) 25:478A (FR-PO469).

[2] George JN Nester CM. Syndromes of thrombotic microangiopathy. N Engl J Med. (2014) 371:654–66. 10.1056/NEJMra1312353 25119611

[3] Meri S. Complement activation in diseases presenting with thrombotic microangiopathy. Eur J Internal Med. (2013) 24:496–502. 10.1016/j.ejim.2013.05.009 23743117

[4] Tsai HM. Untying the knot of thrombotic thrombocytopenic purpura and atypical hemolytic uremic syndrome. Am J Med. (2013) 126:200–9. 10.1016/j.amjmed.2012.09.006 23410558

[5] Markiewski MM Nilsson B Nilsson Ekdahl K Mollnes TE Lambris JD. Complement and coagulation: strangers or partners in crime? Trends Immunol. (2007) 28:184–92. 10.1016/j.it.2007.02.006 17336159

[6] Camous L Roumenina L Bigot S Brachemi S Fremeaux-Bacchi V Lesavre P. Complement alternative pathway acts as a positive feedback amplification of neutrophil activation. Blood. (2011) 117:1340–9. 10.1182/blood-2010-05-283564 21063021

[7] Frimat M Tabarin F Dimitrov JD Poitou C Halbwachs-Mecarelli L Fremeaux-Bacchi V. Complement activation by heme as a secondary hit for atypical hemolytic uremic syndrome. Blood. (2013) 122:282–92. 10.1182/blood-2013-03-489245 23692858

[8] Teoh CW Riedl M Licht C. The alternative pathway of complement and the thrombotic microangiopathies. Transfus Apher Sci. (2016) 54:220–31. 10.1016/j.transci.2016.04.012 27160864

[9] Brocklebank V Wood KM Kavanagh D. Thrombotic microangiopathy and the kidney. Clin J Am Soc Nephrol. (2018) 13:300–17. 10.2215/CJN.00620117 29042465 PMC5967417

[10] Le Clech A Simon-Tillaux N Provot F Delmas Y Vieira-Martins P Limou S. Atypical and secondary hemolytic uremic syndromes have a distinct presentation and no common genetic risk factors. Kidney Int. (2019) 95:1443–52. 10.1016/j.kint.2019.01.023 30982675

[11] Praga M Rodriguez de Cordoba S. Secondary atypical hemolytic uremic syndromes in the era of complement blockade. Kidney Int. (2019) 95:1298–300. 10.1016/j.kint.2019.01.043 31122707

[12] Walport MJ. Complement (Part 2). N Engl J Med. (2001) 344:1140–4. 10.1056/NEJM200104123441506 11297706

[13] Zipfel PF Skerka C. Complement regulators and inhibitory proteins. Nat Rev Immunol. (2009) 9:729–40. 10.1038/nri2620 19730437

[14] Dragon-Durey M-A Loirat C Cloarec S Macher M-A Blouin J Nivet H. Anti-Factor H autoantibodies associated with atypical hemolytic uremic syndrome. J Am Soc Nephrol. (2005) 16:555–63. 10.1681/ASN.2004050380 15590760

[15] Rodriguez de Cordoba S Hidalgo MS Pinto S Tortajada A. Genetics of atypical hemolytic uremic syndrome (aHUS). Semin Thromb Hemost. (2014) 40:422–30. 10.1055/s-0034-1375296 24799305

[16] Loirat C Frémeaux-Bacchi V. Atypical hemolytic uremic syndrome. Orphanet J Rare Dis. (2011) 6:60. 10.1186/1750-1172-6-60 21902819 PMC3198674

[17] Noris M Remuzzi G. Atypical hemolytic-uremic syndrome. N Engl J Med. (2009) 361:1676–87. 10.1056/NEJMra0902814 19846853

[18] Mele C Remuzzi G Noris M. Hemolytic uremic syndrome. Seminars Immunopathol. (2014) 36:399–420. 10.1007/s00281-014-0416-x 24526222

[19] Brocklebank V Johnson S Sheerin TP Marks SD Gilbert RD Tyerman K. Factor H autoantibody is associated with atypical hemolytic uremic syndrome in children in the United Kingdom and Ireland. Kidney Int. (2017) 92:1261–71. 10.1016/j.kint.2017.04.028 28750931 PMC5652378

[20] Bruel A Kavanagh D Noris M Delmas Y Wong EKS Bresin E. Hemolytic uremic syndrome in pregnancy and postpartum. Clin J Am Soc Nephrol. (2017) 12:1237–47. 10.2215/CJN.00280117 28596415 PMC5544502

[21] Noone D Al-Matrafi J Tinckam K Zipfel PF Herzenberg AM Thorner PS. Antibody mediated rejection associated with complement factor H-related protein 3/1 deficiency successfully treated with Eculizumab. Am J Transplant. (2012) 12:2546–53. 10.1111/j.1600-6143.2012.04124.x 22681773

[22] Noris M Remuzzi G. Thrombotic microangiopathy after kidney transplantation. Am J Transplant. (2010) 10:1517–23. 10.1111/j.1600-6143.2010.03156.x 20642678

[23] Sacks SH Zhou W. The role of complement in the early immune response to transplantation. Nat Rev Immunol. (2012) 12:431–42. 10.1038/nri3225 22627861

[24] Ponticelli C. Ischaemia-reperfusion injury: a major protagonist in kidney transplantation. Nephrol Dial Transplant. (2014) 29:1134–40. 10.1093/ndt/gft488 24335382

[25] Rodrigues-Diez R Gonzalez-Guerrero C Ocana-Salceda C Rodrigues-Diez RR Egido J Ortiz A. Calcineurin inhibitors cyclosporine a and tacrolimus induce vascular inflammation and endothelial activation through TLR4 signaling. Sci Rep. (2016) 6:27915. 10.1038/srep27915 27295076 PMC4904742

[26] Jiang X Sung YK Tian W Qian J Semenza GL Nicolls MR. Graft microvascular disease in solid organ transplantation. J Mol Med (Berl). (2014) 92:797–810. 10.1007/s00109-014-1173-y 24880953 PMC4118041

[27] Naesens M Kuypers DR Sarwal M. Calcineurin inhibitor nephrotoxicity. Clin J Am Soc Nephrol. (2009) 4:481–508. 10.2215/CJN.04800908 19218475

[28] Cortina G Trojer R Waldegger S Schneeberger S Gut N Hofer J. De novo tacrolimus-induced thrombotic microangiopathy in the early stage after renal transplantation successfully treated with conversion to everolimus. Pediatr Nephrol. (2015) 30:693–7. 10.1007/s00467-014-3036-8 25577332

[29] Al-Nouri ZL Reese JA Terrell DR Vesely SK George JN. Drug-induced thrombotic microangiopathy: a systematic review of published reports. Blood. (2015) 125:616–8. 10.1182/blood-2014-11-611335 25414441 PMC4304106

[30] Bren A Pajek J Grego K Buturovic J Ponikvar R Lindic J. Follow-up of kidney graft recipients with cyclosporine-associated hemolytic-uremic syndrome and thrombotic microangiopathy. Transplant Proc. (2005) 37:1889–91. 10.1016/j.transproceed.2005.02.112 15919494

[31] Zarifian A Meleg-Smith S O'Donovan R Tesi RJ Batuman V. Cyclosporine-associated thrombotic microangiopathy in renal allografts. Kidney Int. (1999) 55:2457–66. 10.1046/j.1523-1755.1999.00492.x 10354295

[32] Nickeleit V Mengel M Colvin RB. Renal transplant pathology. In:Jennette JC Olson JL Silva FG D'Agati VD, editors. Heptinstall Pathology of the Kidney. 7th ed. Philadelphia, PA: Lippincott Williams & Wilkins (2015). p. 1321–460.

[33] Ponticelli C. *De novo* thrombotic microangiopathy. An underrated complication of renal transplantation. Clin Nephrol. (2007) 67:335–40. 10.5414/CNP67335 17598367

[34] Renner B Klawitter J Goldberg R McCullough JW Ferreira VP Cooper JE. Cyclosporine induces endothelial cell release of complement-activating microparticles. J Am Soc Nephrol. (2013) 24:1849–62. 10.1681/ASN.2012111064 24092930 PMC3810078

[35] Kim YO Lim SW Li C Kang HJ Ahn KO Yang HJ. Activation of intrarenal complement system in mouse model for chronic cyclosporine nephrotoxicity. Yonsei Med J. (2007) 48:517–25. 10.3349/ymj.2007.48.3.517 17594162 PMC2628083

[36] Noone DG Riedl M Pluthero FG Bowman ML Liszewski MK Lu L. Von Willebrand factor regulates complement on endothelial cells. Kidney Int. (2016) 90:123–34. 10.1016/j.kint.2016.03.023 27236750 PMC6591736

[37] Martin-Ramirez J Hofman M van den Biggelaar M Hebbel RP Voorberg J. Establishment of outgrowth endothelial cells from peripheral blood. Nat Protocols. (2012) 7:1709–15. 10.1038/nprot.2012.093 22918388

[38] Riedl M Noone DG Khan MA Pluthero FG Kahr WHA Palaniyar N. Complement activation induces neutrophil adhesion and neutrophil-platelet aggregate formation on vascular endothelial cells. Kidney Int Rep. (2017) 2:66–75. 10.1016/j.ekir.2016.08.015 29142942 PMC5678626

[39] Hyvarinen S Meri S Jokiranta TS. Disturbed sialic acid recognition on endothelial cells and platelets in complement attack causes atypical hemolytic uremic syndrome. Blood. (2016) 127:2701–10. 10.1182/blood-2015-11-680009 27006390

[40] Heinen S Jozsi M Hartmann A Noris M Remuzzi G Skerka C. Hemolytic uremic syndrome: a factor H mutation (E1172Stop) causes defective complement control at the surface of endothelial cells. J Am Soc Nephrol. (2007) 18:506–14. 10.1681/ASN.2006091069 17229916

[41] Barilla-LaBarca ML Liszewski MK Lambris JD Hourcade D Atkinson JP. Role of membrane cofactor protein (CD46) in regulation of C4b and C3b deposited on cells. J Immunol. (2002) 168:6298–304. 10.4049/jimmunol.168.12.6298 12055245

[42] Liszewski MK Atkinson JP. Membrane cofactor protein (MCP; CD46). Isoforms differ in protection against the classical pathway of complement. J Immunol. (1996) 156:4415–21. 10.4049/jimmunol.156.11.4415 8666815

[43] Triantafilou K Hughes TR Triantafilou M Morgan BP. The complement membrane attack complex triggers intracellular Ca^2+^ fluxes leading to NLRP3 inflammasome activation. J Cell Sci. (2013) 126(Pt 13):2903–13. 10.1242/jcs.124388 23613465

[44] Valentijn KM Eikenboom J. Weibel-Palade bodies: a window to von Willebrand disease. J Thromb Haemost. (2013) 11:581–92. 10.1111/jth.12160 23398618

[45] Fiedler U Scharpfenecker M Koidl S Hegen A Augustin HG. The Tie2-ligand angiopoietin-2 is stored in and rapidly released upon stimulation from endothelial cell Weibel-Palade bodies. Cardiovasc Pathol. (2004) 13:495. 10.1016/j.carpath.2004.03.495 14976056

[46] Utgaard JO Jahnsen FL Bakka A Brandtzaeg P Haraldsen G. Rapid secretion of prestored interleukin 8 from Weibel-palade bodies of microvascular endothelial cells. J Exp Med. (1998) 188:1751–6. 10.1084/jem.188.9.1751 9802986 PMC2212514

[47] Noone DG Riedl M Licht C. The role of von Willebrand factor in thrombotic microangiopathy. Pediatr Nephrol. (2017) 33:1297–307. 10.1007/s00467-017-3744-y 28748411

[48] Makou E Herbert AP Barlow PN. Functional anatomy of complement factor H. Biochemistry. (2013) 52:3949–62. 10.1021/bi4003452 23701234

[49] Rodriguez de Cordoba S Esparza-Gordillo J Goicoechea de Jorge E Lopez-Trascasa M Sanchez-Corral P. The human complement factor H: functional roles, genetic variations and disease associations. Mol Immunol. (2004) 41:355–67. 10.1016/j.molimm.2004.02.005 15163532

[50] Schmidt CQ Herbert AP Hocking HG Uhrin D Barlow PN. Translational mini-review series on complement factor H: structural and functional correlations for factor H. Clin Exp Immunol. (2008) 151:14–24. 10.1111/j.1365-2249.2007.03553.x 18081691 PMC2276926

[51] Clark SJ Bishop PN Day AJ. Complement factor H and age-related macular degeneration: the role of glycosaminoglycan recognition in disease pathology. Biochem Soc Trans. (2010) 38:1342–8. 10.1042/BST0381342 20863311

[52] Clark SJ Ridge LA Herbert AP Hakobyan S Mulloy B Lennon R. Tissue-specific host recognition by complement factor H is mediated by differential activities of its glycosaminoglycan-binding regions. J Immunol. (2013) 190:2049–57. 10.4049/jimmunol.1201751 23365078 PMC3672945

[53] Kazatchkine MD Fearon DT Austen KF. Human alternative complement pathway: membrane-associated sialic acid regulates the competition between B and β1H for cell-bound C3b. J Immunol. (1979) 122:75–81. 10.4049/jimmunol.122.1.75 762425

[54] Schmidt CQ Herbert AP Kavanagh D Gandy C Fenton CJ Blaum BS. A new map of glycosaminoglycan and C3b binding sites on factor H. J Immunol. (2008) 181:2610–9. 10.4049/jimmunol.181.4.2610 18684951

[55] Zaferani A Vives RR van der Pol P Navis GJ Daha MR van Kooten C. Factor H and properdin recognize different epitopes on renal tubular epithelial heparan sulfate. J Biol Chem. (2012) 287:31471–81. 10.1074/jbc.M112.380386 22815489 PMC3438980

[56] Yang L Guan H He J Zeng L Yuan Z Xu M. VEGF increases the proliferative capacity and eNOS/NO levels of endothelial progenitor cells through the calcineurin/NFAT signalling pathway. Cell Biol Int. (2012) 36:21–7. 10.1042/CBI20100670 21895605

[57] Liptak P Ivanyi B. Primer: Histopathology of calcineurin-inhibitor toxicity in renal allografts. Nat Clin Practice Nephrol. (2006) 2:398–404. 10.1038/ncpneph0225 16932468

[58] Seino J Eveleigh P Warnaar S van Haarlem LJ van Es LA Daha MR. Activation of human complement by mouse and mouse/human chimeric monoclonal antibodies. Clin Exp Immunol. (1993) 94:291–6. 10.1111/j.1365-2249.1993.tb03446.x 8222320 PMC1534236

[59] Schiff J Cole E Cantarovich M. Therapeutic monitoring of calcineurin inhibitors for the nephrologist. Clin J Am Soc Nephrol. (2007) 2:374–84. 10.2215/CJN.03791106 17699437

[60] Basu A Datta D Zurakowski D Pal S. Altered VEGF mRNA stability following treatments with immunosuppressive agents: implications for cancer development. J Biol Chem. (2010) 285:25196–202. 10.1074/jbc.M110.119446 20554520 PMC2919082

[61] Bouvier N Flinois JP Gilleron J Sauvage FL Legendre C Beaune P. Cyclosporine triggers endoplasmic reticulum stress in endothelial cells: a role for endothelial phenotypic changes and death. Am J Physiol Renal Physiol. (2009) 296:F160–9. 10.1152/ajprenal.90567.2008 18987109

[62] Farivar AS Mackinnon-Patterson BC Barnes AD McCourtie AS Mulligan MS. Cyclosporine modulates the response to hypoxia-reoxygenation in pulmonary artery endothelial cells. Annals Thoracic Surg. (2005) 79:1010–6. 10.1016/j.athoracsur.2004.08.078 15734424

[63] Gooch JL King C Francis CE Garcia PS Bai Y. Cyclosporine a alters expression of renal microRNAs: new insights into calcineurin inhibitor nephrotoxicity. PLoS ONE. (2017) 12:e0175242. 10.1371/journal.pone.0175242 28414804 PMC5393575

[64] Grupper A Shashar M Bahry D Pri-Paz Y Ben Tur O Levi S. Cyclosporine attenuates arginine transport, in human endothelial cells, through modulation of cationic amino acid transporter-1. Am J Nephrol. (2013) 37:613–9. 10.1159/000350614 23796541

