# Supplementary material for: The loss of glycocalyx integrity impairs complement factor H binding and contributes to cyclosporine-induced endothelial cell injury
Source: Front Med (Lausanne). 2023 Feb 13;10:891513. doi: 10.3389/fmed.2023.891513 (PMC9968885; doi:10.3389/fmed.2023.891513)

SUPPLEMENTAL FILE

TABLE OF CONTENTS

MATERIALS AND METHODS

- Antibodies for flow cytometry
- Reagents and antibodies for CFH surface cofactor activity assay
- Antibodies for immunofluorescence

FIGURES

- Supplemental Figure 1
- Supplemental Figure 2
- Supplemental Figure 3
- Supplemental Figure 4
- Supplemental Figure 5

MATERIALS AND METHODS

Antibodies for flow cytometry

For detection of complement deposition by flow cytometry, we used a rabbit polyclonal anti-C3c antibody directly conjugated with FITC (abcam, Cambridge, UK, ab4212); and goat anti-C9, 1:100 dilution (Complement Technology Inc., TX, USA, A226). Anti-goat secondary Alexa Fluor 488 antibody was used at a dilution of 1:200.

For detection of complement regulators, we used rabbit anti-CD46, 1:50 dilution (flow cytometry) and 1:500 dilution (Western blot) (Santa Cruz Biotechnology, Dallas, TX, USA, sc-9098); goat anti-CD55, 1:100 dilution (flow cytometry) and 1:2000 dilution (Western blot) (R&D Systems, Minneapolis, USA, AF2009); and rat anti-CD59, 1:100 dilution (flow cytometry) and 1:1000 dilution (Western blot) (AbD Serotec, Oxford, UK, MCA715G). Mouse-anti-glyceraldehyde 3-phosphate dehydrogenase (GAPDH), 1:2000 dilution (EMD Millipore, MAB374) was used as loading control in Western blots. Corresponding species-specific Alexa Fluor 488 antibodies were used at a dilution of 1:200 for flow cytometry and species-specific secondary HRP-conjugated antibodies were used at a dilution of 1:2000 for anti-CD46 and anti-CD59; dilution of 1:3000 for anti-CD55; and dilution of 1:5000 for anti-GAPDH. Fixable Viability Dye eFluor780 (eBioscience, San Diego, CA, USA, 1:1000 dilution reconstituted in PBS) was used to label deal cells for flow cytometry.

For detection of complement factor H (CFH) binding by flow cytometry, we used purified CFH (CSL Behring, Marburg, Germany) that was tagged with Alexa Fluor 488 (concentration 2 mg/ml). For each 100 µl of cell suspension, we added 2 µl (4 µg) of Alexa Fluor 488-tagged CFH.

Reagents and antibodies for complement factor H surface cofactor activity assay

For CFH surface cofactor activity assays, we used purified complement factor H (CSL Behring, Marburg, Germany) at a concentration of 10 μg/ml, complement factor I (EMD Millipore Corp., MA, USA, 341280) at a concentration of 10 μg/ml, and C3b (EMD Millipore Corp., MA, USA, 204860) at a concentration of 3.3 μg/ml. For detection of C3 on Western blot, we used goat anti-C3, 1:1000 dilution (Complement Technology Inc., TX, USA, A213) with corresponding anti-goat secondary HRP-conjugated antibody at a dilution of 1:5000.

Antibodies for immunofluorescence

For BOEC glycocalyx imaging, we used an Alexa Fluor 594-conjugated wheat germ agglutinin (Thermo Fischer Scientific, W11262, dilution 1:500), mouse anti-heparan sulfate (Abcam, Cambridge, UK, ab23418, dilution 1:100), peanut agglutinin (Vector Labs, Ontario, CA, FL-1071-5, dilution 1:200).

For Weibel-Palade body release of VWF, we used a rabbit anti-VWF (DakoCytomation, A0082, dilution 1:100).

Supplemental Figure 1:

Cyclosporine causes dose- and duration-dependent endothelial cell cytotoxicity.

Blood outgrowth endothelial cell (BOEC) cytotoxicity detected using an LDH assay. Increasing cyclosporine (CsA) dose and duration of exposure resulted in increased BOEC cytotoxicity

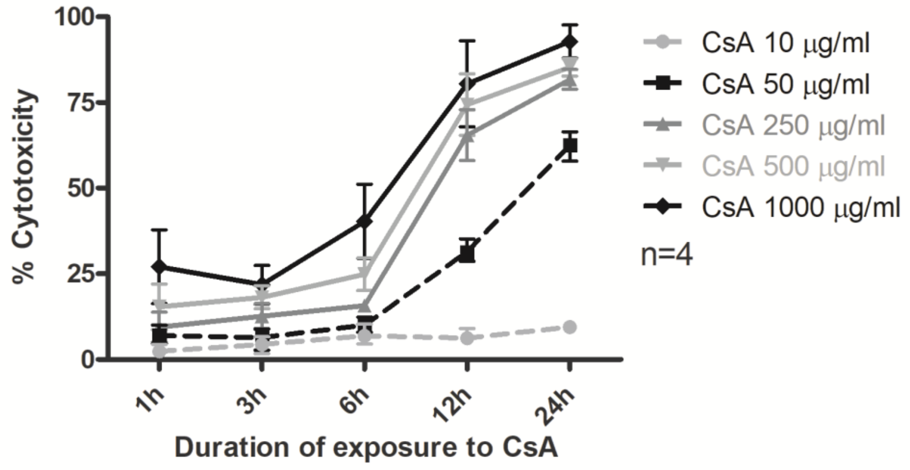

Supplemental Figure 2:

Cyclosporine causes endothelial cell release of Weibel-Palade bodies and its contents.

Von Willebrand factor (VWF) release from blood outgrowth endothelial cells (BOECs) was detected via immunofluorescence. Control BOECs were treated with media for 24 hours, with or without further incubation with 50% normal human serum (50% NHS) for 30 minutes. BOECs treated with media for 24 hours, followed by incubation with anti-CD59 antibody for 30 minutes, and subsequent 50% NHS for a further 30 minutes was used as positive control. The experimental condition was with BOECs treated with cyclosporine (CsA) 10 µg/ml for 24 hours. BOECs were fixed with 2% paraformaldehyde and permeabilized with 0.2% Triton in phosphate-buffered saline, followed by incubation with rabbit anti-VWF (green) and goat anti-VE-cadherin (red). Images were taken using an IX81 inverted microscope (Olympus Corp., Tokyo, Japan) with a 60/1.35 oil immersion objective and a C9100-13 back-thinned EM-CCD camera (Hamamatsu Photonics, Hamamatsu City, Shizuoka Pref., Japan) with a CSU X1 spinning disck confocal scan head (Yokogawa, Yokogawa Canada Inc., AB, Canada). Bar = 22 µm. Treatment with CsA 10 µg/ml for 24 hours led to less intracellular VWF and less intense staining of VE-cadherin (E-F).

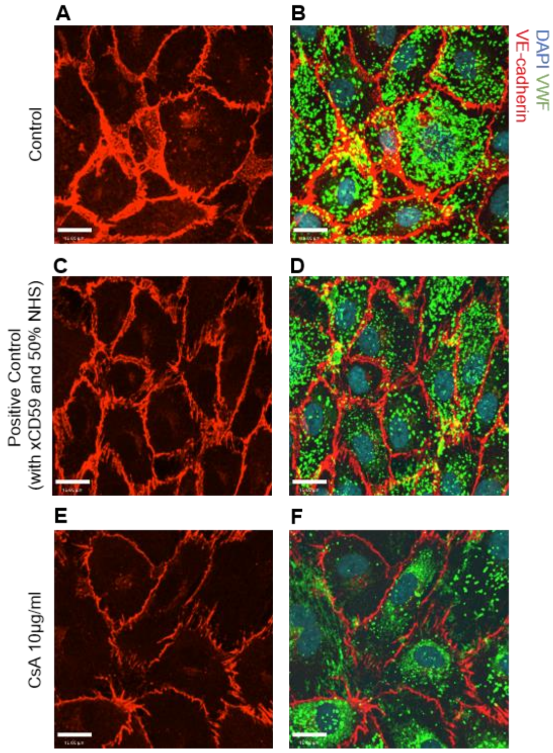
A
B
C
D
E
F
Control
Positive Control
(with xCD59 and 50% NHS)
CsA 10µg/ml
DAPI VWF
VE-cadherin

Supplemental Figure 3:

Cyclosporine led to impaired complement factor H (CFH) surface cofactor activity

CFH surface cofactor activity assay. Blood outgrowth endothelial cells (BOECs) were incubated with C3b 3.3 μg/ml and CFI 10 μg/ml at 37 degrees Celsius, with or without pre-incubation with CFH 10 μg/ml at 37 degrees Celsius. After the various time points, the supernatant was inactivated in sample buffer and separated by SDSPAGE and transferred onto a nitrocellulose membrane, and the appearance of C3b degradation fragments were analysed by Western blotting (representative Western blots are shown in figures). A) Endogenous cofactor activity on BOEC without CFH. BOECs were incubated with C3b and CFI at 37 degrees Celsius. Degradation products (**α**'68, **α**'46 and **α**'43 kDa fragments of the C3b **α**' chain) were detectable after 90 minutes and increased with time. There were no significant differences between the endogenous cofactor activity on BOEC without CFH whether they were exposed to A) media, C) Neuraminidase, E) cyclosporine (CsA) 10 μg/ml, G) CsA 50 μg/ml, or I) CsA 100 μg/ml. B) Cofactor activity of CFH on surface of BOEC. BOECs were preincubated with CFH for 1 hour at 37 degrees Celsius and thoroughly washed, prior to incubation with C3b and CFI at 37 degrees Celsius. Degradation products were detectable after 15 minutes. D) Cofactor activity of CFH on surface of neuraminidase-treated BOEC. Neuraminidase cleaves sialic acid groups from cell surfaces. BOECs were pre-incubated with neuraminidase 500 mU/ml for 1 hour followed by CFH 10 μg/ml for 1 hour at 37 degrees Celsius, prior to being thoroughly washed and incubated with C3b 3.3 μg/ml and CFI 10 μg/ml at 37 degrees Celsius. Degradation products were detectable after 60 minutes. F, H, J) Cofactor activity of CFH on surface of CsA-treated BOEC. BOECs were pre-incubated with CsA 10 μg/ml, CsA 50 μg/ml or CsA 100 μg/ml for 24 hours. They were then incubated with CFH 10 for 1 hour at 37 degrees Celsius, washed thoroughly, and then incubated with C3b and CFI at 37 degrees Celsius. After the various time points, the supernatant was inactivated in sample

buffer and separated by SDSPAGE and transferred onto a nitrocellulose membrane, and the appearance of C3 fragments was analysed by Western blotting. Degradation products were detectable: F) CsA 10 μg/ml after 45 minutes, H) CsA 50 μg/ml after 45 minutes, and J) CsA 100 μg/ml after 90 minutes.

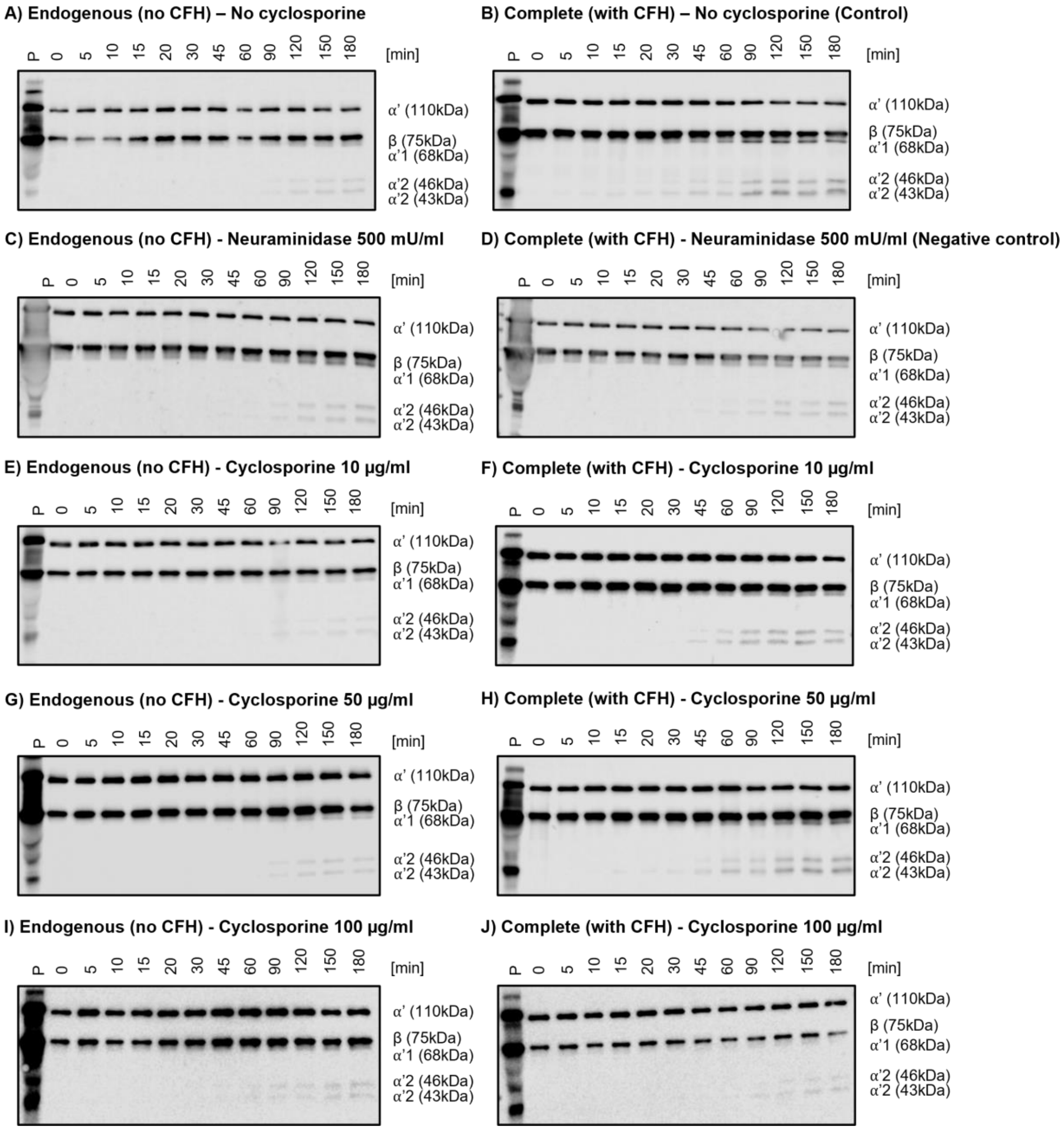

Supplemental Figure 4:

Cyclosporine led to decreased heparan sulfate and CFH

Heparan sulfate and CFH on blood outgrowth endothelial cell (BOEC) surface was detected by immunofluorescence. BOECs were incubated in media (control) or cyclosporine (CsA) 10 μg/ml for 24 hours. Treatment with heparinase III 0.5 U/ml for 30 min, which degrades heparan sulfate was used as positive control. Representative images (A-L) and mean fluorescence intensity from 3 sets of experiments with 10 representative images taken per condition (each dot represents 1 image) were measured with ImageJ and summarized (M-N). BOEC treated with CsA had decreased heparan sulfate and CFH compared to that of control BOEC (n=3, ***$p<0.0001$, paired two-tailed t-test). As expected, BOEC treated with heparinase III also had decreased heparan sulfate and CFH.

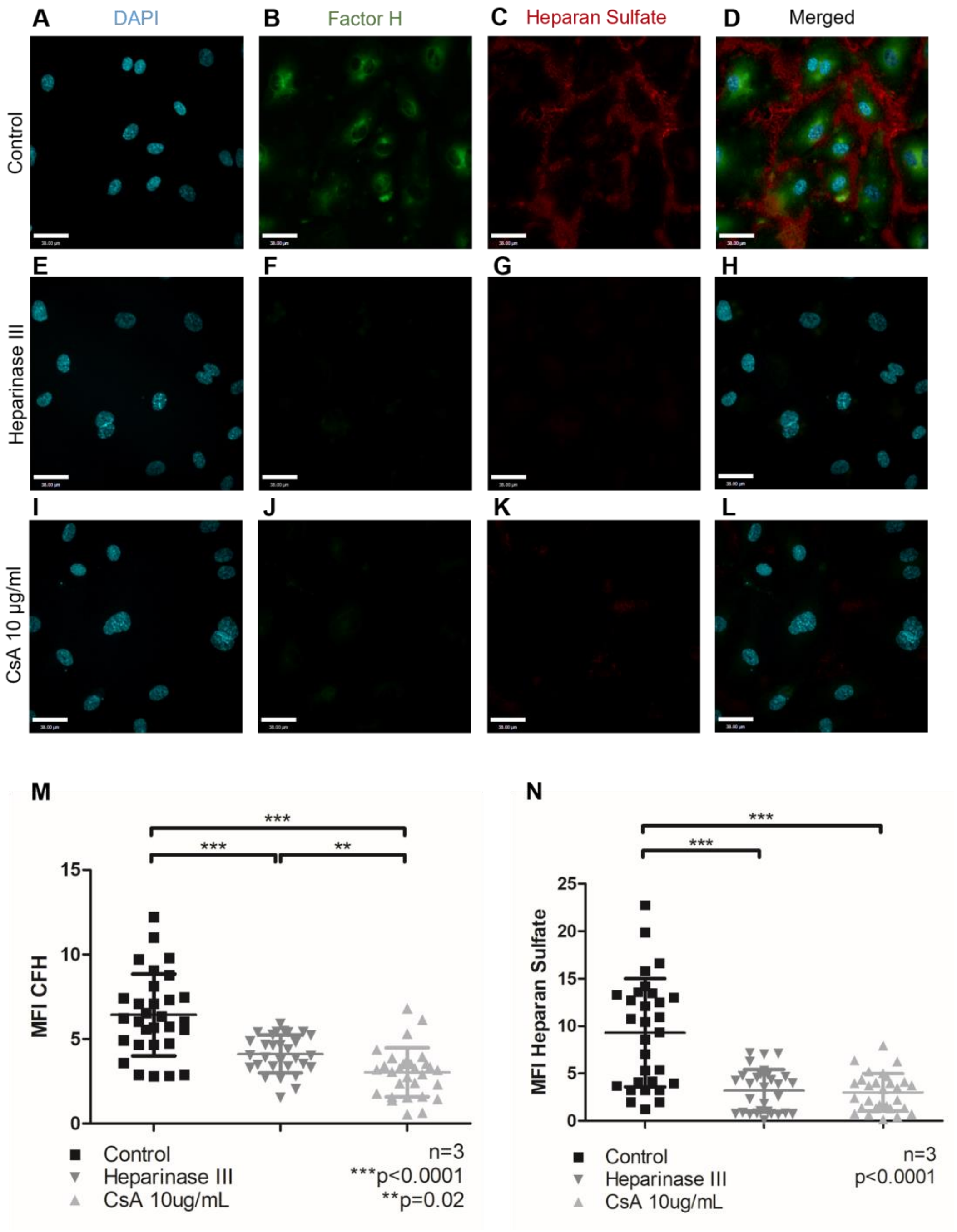
A DAPI
B Factor H
C Heparan Sulfate
D Merged
Control
E
F
G
H
Heparinase III
I
J
K
L
CsA 10 µg/ml
M
MFI CFH
***
***
**
15
10
5
0
Control
Heparinase III
CsA 10ug/mL
n=3
***p<0.0001
**p=0.02
N
MFI Heparan Sulfate
***
***
25
20
15
10
5
0
Control
Heparinase III
CsA 10ug/mL
n=3
p<0.0001

Supplemental Figure 5:

Cyclosporine did not lead to loss of sialic acid.

Peanut agglutinin (PNA) on blood outgrowth endothelial cell (BOEC) surfaces was assessed by flow cytometry. BOECs were incubated in media (control) or cyclosporine (CsA) 10 µg/ml for 24 hours. Treatment with neuraminidase 500 mU/ml, which cleaves sialic acid groups from glycoproteins was use as positive control. Non-viable cells were excluded from analysis with Fixable Viability Dye eFluor 780. Representative histogram (A) and graphical summary of $\log_{10}$ mean fluorescence intensity (MFI) from experiments (B). The $\log_{10}$ MFI of PNA in BOECs treated with CsA 10 µg/ml was similar to that of control. As expected, treatment with neuraminidase 500 mU/ml led to increased PNA (n=3, ***p=0.005, paired two-tailed t-test).

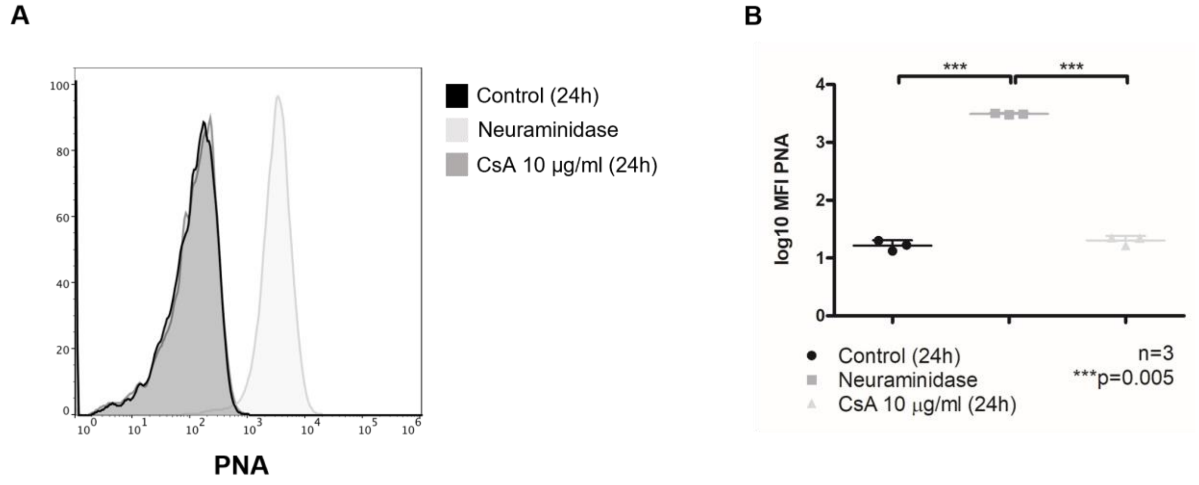

Supplement: Supplementary file 1 [file Data_Sheet_1.pdf]
